# Cardiac Output Monitoring: Validation Studies–how Results Should be Presented

**DOI:** 10.1007/s40140-017-0239-0

**Published:** 2017-10-27

**Authors:** Peter M. Odor, Sohail Bampoe, Maurizio Cecconi

**Affiliations:** 1 Department of Anaesthesia, St. George’s University Hospital, London, SW17 0QT UK; 2 Centre for Perioperative Medicine, University College London, London, UK; 3 Department of Anaesthesia and Perioperative Medicine, University College Hospital, London, UK; 4 Department of Anaesthesia and Intensive Care, St. George’s University Hospital, London, UK

**Keywords:** Bland-Altman analysis, Accuracy, Precision, Cardiac output, Method comparison, Hemodynamic monitoring

## Abstract

**Purpose of Review:**

Cardiac output monitors can be assessed by a variety of techniques, but a common principle is quantifying agreement between a reference standard and new monitor. The current standard analysis technique is a Bland-Altman plot. The Bland-Altman plot evaluates bias between mean differences of cardiac output, from which an agreement interval is derived. These limits are, however, statistical limits of agreement and the clinical acceptability will depend upon context and application. This article provides suggestions for understanding and presenting the results of cardiac output validation, using standard metrology alongside proposals for criteria used to accept new techniques.

**Recent Findings:**

Confusion about the appropriate way to report “precision” in method comparison studies stem from a lack of clarity on how single or repeated measurements should be interpreted. During serial measurements of cardiac output the true value changes, thus measurement should be considered as serial rather than repeated. Method agreement based upon precision achieved by cardiac output monitors needs to consider each method’s general variability around true values obtained and this data should be generated and presented as part of each study design.

**Summary:**

Studies should report serial measurements from two techniques for cardiac output monitoring. Results of similar techniques from other studies may not always be transferred and compared. Bias and intervals of agreement should be presented as Bland-Altman plots with dynamic cardiac output trends in polar plots. Percentage error should be calculated to allow appropriate comparison of techniques for study populations with different expected cardiac output values.

## Introduction

Validation studies of cardiac output monitoring involve statistical comparison of different techniques for the measurement of equivalent physiological parameters. Cardiac output (CO) plays a crucial role in the haemodynamic management of critically ill patients treated in the intensive care unit and in surgical patients undergoing major surgery [1, 2]. Innovative measurement tools for CO determination are becoming increasingly available and frequently less invasive, with a corresponding increase in studies comparing these techniques [2–6, 7•, 8–10]. Deciding which tool is most appropriate to use in clinical settings requires an understanding of comparison studies, so that their inherent measurement properties can be balanced alongside practical implications, costs and risks.

Fair and valid comparison of CO monitoring devices requires not only robust and sensitive methods for performing the studies, but also similarly rigour applied to the analysis techniques and presentation of results. Various formats for data reporting exists, variably including different descriptions, with a lack of consistency in how the results of bias and precision statistics should be presented. This article provides suggestions for understanding and presenting the results of cardiac output validation research, using standard metrology alongside proposals for criteria used to accept new techniques.

## Method Comparison

To evaluate a new technique of CO monitoring we must compare resultant measurements with a known reference standard. It is vital to understand that heart rate and stroke volume vary rapidly in response to pathophysiological conditions and cyclically in association with physiological changes, such as respiration. As such, the true CO varies dynamically and therefore the reference standard is expected to be serial measurements of multiple, changing values. This is still true for monitoring techniques that report “continuous” CO, despite averaging outputted value over a few seconds to minimise the effect of respiration. Thus variability exists within in variable measurement, which makes comparison studies more difficult to perform. Several reference standards are used in CO monitoring validation studies, since unfortunately there is no ideal reference standard that meets all the ideal criteria of quality. Such quality criteria include knowledge of the true precision of the monitoring system, the physiological intra-patient variability of the measured variable, the inter-patient variability, inter-device variability and presence of minimal measurement artefacts. In place of an ideal reference standard, we must instead use the best comparable reference standard. In this context, the most commonly applied reference standard for CO monitoring is an averaged set of single-indicator transpulmonary thermodilution curves taken from a pulmonary artery catheter [11, 12]. This measurement technique, based upon the Fick principle [13], has been extensively studied and the level of precision is well described.

## Accuracy and Precision

In simple terms, methods of CO monitoring may be compared by reference to two measurement outcomes: (1) accuracy and (2) precision. Accuracy describes the systematic error of the measurement tool and is defined as how close the measured value is to the true value. Precision describes the reproducibility of measurements; otherwise, considered as the variability of repeated values due to random error. A measurement tool may be precise but inaccurate, meaning that resultant values are consistent, but similarly far from the true value. Clearly it is preferable for a CO measurement tool to be both precise and accurate, meaning that systematic and random errors inherent in the tool are low. A measurement device with high accuracy has a low bias, meaning that the arithmetic mean of all differences in measurements between the tool values and true values is low. The metrology of measurement is more complex [14], but a clear understanding of the above two concepts is sufficient to appreciate the key concepts in method comparison.

## “Bland-Altman” Plot

Correlation studies assess the relationship between one variable and another, not the differences. Therefore, correlations and regression studies can be misleading and are not appropriate as a method for assessing the comparability between CO measurement methods. Instead, the current recognised standard statistical method of assessing agreement between two serial measurements of the same clinical variable is the “Bland-Altman” plot [15•, 16, 17]. The Bland-Altman plot is a simple, graphical way to illustrate bias between mean differences and to estimate a proportion of agreement for the two measurement methods.

The Bland-Altman plot should be presented as a scatter plot in which the x axis represents the average of a pair of measurements (A + B/2), and the *y* axis shows the difference between the two paired measurements (A − B). The *x* axis of a Bland-Altman plot for CO measurement displays the arithmetic mean CO output (in L/min, for example) of a pair of values taken at the same time point using the reference standard and new measurement tool. The *y* axis for each point is plotted as the difference in CO values (in L/min) for that same paired data set at the same time point. The Bland-Altman plot allows visual inspection for several aspects of the comparability of measurement methods. First, a consistent measure of bias can be described. This is the arithmetic mean of all the differences in paired measurement between tools and is represented as a line across the *x* axis of the plot, with the difference between this value and a *y* value of 0 describing the magnitude and direction of the bias. Bias can be reported in absolute terms or as a percentage (bias/mean value). A bias of close to zero describes a new measurement tool with high accuracy, assuming that the reference tool is the same as the true CO value. Of course, the Bland-Altman plot may also show data points scattered throughout the chart, well above and below the zero point on the *y* axis. Such presentation may suggests that there is no consistent bias of one measurement tool versus the other, but does not exclude hidden or inconsistent bias, and is an inherent limitation of using Bland-Altman analysis alone.

The limits of agreement are the plotted lines within which 95% of all the points fall on either side of the bias (that is, ± 1.96 × the standard deviation around the bias). Limits of agreement refer to precision of the measurement tool, so if the limits are narrow then the precision is high and if the limits are wide then the precision is low. The ideal result for a CO measurement technique is for a very small bias with tight limits of agreement. However, the limits of agreement may only be interpreted properly if the confidence intervals for the limits are known [18, 19]. Unfortunately such confidence intervals are consistently poorly presented by studies using Bland-Altman plots [20, 21]. Variability in the data structure is expected to be higher when small numbers of readings from large numbers of patients are taken, rather than many readings from a single patient. We suggest that the data structure (namely whether recorded COs are single paired measurements, replicates or several measures in different subjects) and confidence intervals for the limits of agreement should both be reported in validation studies, consistent with recent conclusions elsewhere [22••].

**Fig. 1:**
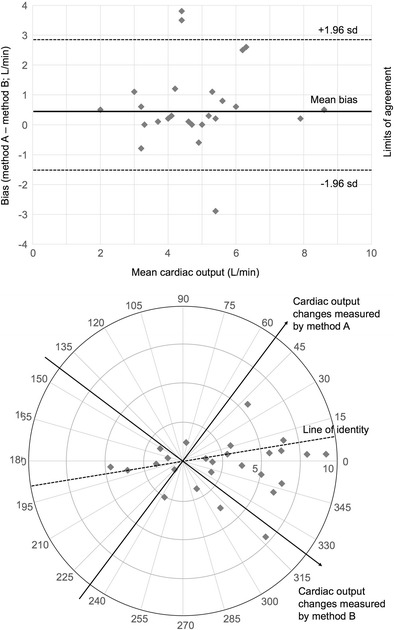
Bland-Altman plot and polar plots for new techniques versus reference techniques, with representation of the limits of agreement and lines of identity respectively (dotted lines)

The Bland-Altman plot can also describe how the magnitude of the measured value influences differences in the two measurement methods. For example, low CO states may generate larger differences in results for two measurement tools than at higher CO states. This can be identified as differences in the mean differences that are more apparent at one end of the plot, showing, for example, a tool that consistently over-estimates high values or under-estimates low values. See Fig. 1 for an example Bland-Altman plot and how this compares with a polar plot.

## Percentage Error

The Bland-Altman plot does not state if the limits of agreement are sufficient to justify the suitable use of a clinical measurement device; such limits should be defined a priori and are related to the population being studied and inherent error within both studied methods of CO measurement. For example, a limit of agreement of ± 1 L/min may be acceptable in patients with a high mean CO of 10 L/min, but may not be acceptable in a paediatric population with a much lower mean CO. The solution to this problem is suggested to be reporting the percentage error (PE) of the limits of agreement, which can be used as a cut-off for whether to accept a new technique. The PE therefore makes understanding CO study results context-sensitive, even gives different study population with different expected CO values.

PE is calculated by dividing the limits of agreement by the mean value of measurements taken using the reference method of CO monitoring in the required population [23••]. A PE cut of ± 30% has been suggested as a pragmatic guide for clinicians to determine whether a new measurement technique represents a good alternative to the reference standard. The basis of this approach is that the level of precision should be at least equivalent to the reference standard i.e. thermodilution, which is ± 20%. Since random error in measurement is compounded during combination of two precisions, with two measurements at ± 20% equating to a total error of ± 28.3%, the total PE is commonly rounded to ± 30%. Thus, finding a percentage error of less than ± 30% equates to the new tested technique having an error similar to the reference standard, which should therefore be considered acceptable.

The limitation to the commonly held assumption on combined errors in PE is that the precision of thermodilution technique can vary, depending upon the technique used. If the technique is applied rigorously and the error used for the thermodilution technique is lower than ± 20%, then this may inappropriately lead to acceptance of a new measurement technique. In this situation, the combined error may still be lower than ± 30%, if a lower than expected error for the reference standard compensates for an error of higher than ± 20% in the tested measurement technique. Cecconi et al. demonstrated that by changing the error of the reference technique, by averaging different numbers of measurements, the overall agreement can significantly change. In practice, the error of the reference technique plays a very significant factor when testing the agreement of a new technique [24••]. Therefore, we suggest that the actual precision of the reference technique within a study of CO monitoring devices should be measured and reported, alongside the combined PE. Le Manach and Collins [25] confirmed this by performing a set of simulations in which they compared an almost “perfect” device with zero bias (perfect accuracy) and 4% precision to a reference device with different levels of precision.

## Precision of Method

As previously described, a commonly used statistical construct for method comparison is an assessment of both accuracy of agreement (bias) and precision of agreement using Bland-Altman analysis. Correct interpretation of a Bland-Altman plot will result in either acceptance of rejection of the inter-changeability of the two methods being compared. A degree of caution must be exercised, however, when Bland-Altman plots are interpreted and the precision of method must be considered before conclusions are formed. For true agreement, bias should be zero and the precision of agreement should be as high as possible. It should be noted that precision of agreement is influenced by the precision of the methods being compared. Any measurement technique in which an unknown variable (such as cardiac output) is being estimated may be prone to error, even when compared to itself. It is important to take this imprecision into account when interpreting measurements of agreement between two differing methods because significant imprecision of method in either method being compared will contribute to worse precision of agreement.

## Trend Analysis

CO monitors may be used to estimate absolute values for cardiac output. Bland-Altman analysis can show measurement agreement between various measurement methods. However, in clinical practice, modern cardiac output monitors are commonly used for continuous measurement of cardiac output, or trend analysis. Bland-Altman plots allow a comparison of how well the studied technique agrees with the reference technique but may not be the best method for analysing trend data because trend data analysis should involve analysis of the change in cardiac output, or ΔCO, instead of absolute CO [26••]. In clinical practice, the use of CO monitors as trend monitors is commonplace and an appropriate statistical method for analysing trending ability should be selected when presenting comparisons between technologies. There has been some debate over the most appropriate method for statistical analysis. Critchley et al. [27••] performed a critical review of published articles that compared methods of continuous CO measurement, finding that less than one fifth of published studies compared trending ability. Of those that did, a variety of different methods were used including Bland-Altman analysis of histograms and tables, time plots, regression analysis of scatter plots and analysis direction of change as a statistic [27••].

The precision of a device is very important to understanding how to interpret changes reported by the device. The least significant change (LSC) derived as 2 × √2 × (standard error of the mean) is an important variable as any change below this should not be considered as a real change. This can be useful also to identify in a comparison study which pairs of data do not contribute to the comparison of real changes in CO. The thresholds can be used to identify pairs of data that do not contribute to a real trend analysis, as no change above the LSC has occurred.

Bland-Altman analysis fundamentally relies on the assumption that the data points being compared are unrelated. This cannot be true for clinical studies of trending ability for continuous CO monitors because repeated measurements must, and do come from the same subject [15•, 26••]. The resulting underestimation of true variability can be corrected for, but only in terms of precision rather than trending [26••].

Twenty-three of the studies appraised in the Critchley et al. review [26••] used the Cartesian technique of plotting paired readings of ΔCO (reference technique) and ΔCO (studied technique) and performed a concordance analysis. Such concordance analysis relies on the direction of change of cardiac output as a statistic and somewhat ignores the magnitude of that change. Critchley et al. proposed a novel method of using polar plots as a visual representation for trending comparison. Data are presented in four-quadrant plots in a similar way to plots used for Bland-Altman analysis; however, polar plots present data points radially about a polar origin, where accuracy of agreement is represented by the mean of the polar angles formed by those data points, and the length of the radius can reflect the mean value of ΔCO. [26••] A modified concordance analysis can then be performed using predefined radial limits (rather than the *x* and *y* axes used in Cartesian methodology) [26••]. An example polar plot can be seen in Fig. 1. Polar plots can therefore be used to compare trending ability of different methods of measuring CO in validation studies.

## Conclusion

A multitude of techniques have been recently used to report the outcomes of CO validation studies but, much like a shift from static picture to moving image, the preferred approach is now reporting of dynamic trend analysis. Bland-Altman plot analysis allows for clarity and presentation of method agreement based upon general variability of static CO around true values, allowing precision of method to be described. The data structure for CO validation studies contain multiple potential layers of variability, including beat-to-beat recorded variability within patients, variability causes by differing ratios of CO readings per patient and total patients included. A requirement of this data complexity is a need to provide true representation of results, including declaration of a priori limits of agreement and of post hoc confidence intervals. Interpretation of Bland-Altman plot limits of agreement requires appreciation that these are statistically rather than clinically derived parameters. Polar plots present ΔCO trend data and are a better fit with contemporary clinical use, which tends to be more concerned with dynamic patterns of CO trends following fluid interventions, rather than the absolute values. Acceptance of a CO monitor measurement precision requires correlation with clinical applicability for the range of CO expected in the patient population that the device will be used in. Percentage error should be calculated to allow appropriate comparison of techniques for study populations with different expected CO values. There is no universal mandatory standard of reporting for CO monitor validation studies, but a recent systematic review of Bland-Altman studies suggested a list of key features for adequate presentation of data [22••]. A formal list of reporting criteria would aid standardisation of CO validation study reporting and enable improved future meta-analysis.
